# Central statistical monitoring in multicentre clinical trials: developing statistical approaches for analysing key risk indicators

**DOI:** 10.1186/1745-6215-14-S1-P139

**Published:** 2013-11-29

**Authors:** Elsa Valdes-Marquez, Jemma C Hopewell, Jane Armitage, Martin Landray

**Affiliations:** 1 Clinical Trial Service Unit and Epidemiological Studies Unit, University of Oxford, Oxford, UK

## Objective

Central statistical monitoring (CSM) can identify trial misconduct, and help to prioritise on-site visits and additional training. Key risk indicators (KRIs) focus CSM on variables most likely to affect study reliability or patient safety. We developed the use of robust minimum covariance determinant (MCD) distances to detect outlying centres in the context of KRI analyses.

## Method

Initially, a summary statistic (e.g. mean) describing the KRI is calculated for each centre and robust MCD-based distances are calculated by the FAST-MCD algorithm (Rousseeuw, 1999). For each KRI, robust estimates of the multivariate location  and scatter  are defined by minimizing the determinant of the covariance matrix and generalized distances, , are calculated. The distances follow a  distribution (where n is the multivariate dimension) and define corresponding p-values. Outliers are defined as centres with p-values below a pre-defined threshold (e.g. p<0.05). This method can be used for univariate and multivariate KRIs. Furthermore, p-values can be combined across KRIs to form a single score. We provide empirical examples and graphical displays of univariate and multivariate KRI analyses undertaken using this robust MCD distance approach based on a large-scale multicentre cardiovascular trial.

## Conclusion

Robust MCD distances offer a flexible approach for analysing both univariate and multivariate KRIs and can be implemented in standard statistical software.

